# Triple Anesthesia: Combining Sevoflurane, Propofol, and Remimazolam for General Anesthesia in a Case Series

**DOI:** 10.7759/cureus.66737

**Published:** 2024-08-12

**Authors:** Rebecca Koch, Richard Witkam, Lucas T van Eijk, Jörgen Bruhn

**Affiliations:** 1 Anesthesiology, Radboud University Medical Center, Nijmegen, NLD; 2 Anesthesiology, Radboud University Medical Center,, Nijmegen, NLD

**Keywords:** monitoring, general anesthesia, sevoflurane, propofol, remimazolam, triple anesthesia

## Abstract

There is an ongoing quest for an *ideal* uniform anesthesia regimen that adequately covers all nociceptive stimuli preventing hypertension and tachycardia while minimizing hypotension and the need for antihypotensive drugs. Recently, the ultra-short-acting benzodiazepine remimazolam was approved for the induction and maintenance of general anesthesia. Combining remimazolam with sevoflurane and propofol may combine the antiemetic properties of propofol, the depressing (immobilizing) effect on spinal motor neurons of sevoflurane, and the hemodynamic stability afforded by remimazolam, making it an attractive addition to the armamentarium of anesthetic agents. We describe five patients in whom general anesthesia was maintained with this triple combination, along with multimodal analgesia. All patients maintained hemodynamic stability at sufficient hypnotic depth, with no observable movement during surgery or episodes of cardiac arrhythmias.

## Introduction

There is an ongoing quest for an *ideal* anesthesia regimen. For minimal intervention anesthesia, it would be advantageous to have a standard uniform anesthesia approach that adequately covers all nociceptive stimuli preventing hypertension and tachycardia while minimizing hypotension and the need for antihypotensive drugs. The need for intraoperative interventions such as extra boluses of a shorter-acting opioid or repetitively increasing or decreasing the dosing of continuously administered hypnotic drugs should be minimal or even zero. The common postoperative problems of acute postoperative pain and postoperative nausea and vomiting (PONV) should be addressed with multimodal analgesia components and low intraoperative opioid use to prevent opioid-induced hyperalgesia and the integration of anesthetic drugs with antiemetic properties. Here, we report the combined use of the hypnotic drugs sevoflurane, propofol, and remimazolam next to a multimodal analgesia concept.

Remimazolam, a short-acting benzodiazepine, was recently approved for the induction and maintenance of general anesthesia in several countries. One of its main advantages for use in general anesthesia is the favorable hemodynamic stability. For maintenance of general anesthesia, it can be given as the only hypnotic component at a relatively high dose (1-2 mg/kg/hour) in combination with a relatively high dose of remifentanil and complete muscle relaxation to prevent hemodynamic or motor reaction [1]. Premedication with an oral benzodiazepine such as midazolam is broadly used and adds considerable efficacy to other hypnotic drugs at least during induction of anesthesia. However, short-acting remimazolam is the only benzodiazepine that can be added continuously during the entire surgery without prolonging the time to awakening inadequately. The advantages of the combination of propofol and a volatile anesthetic for general anesthesia have been described [2]. We report the use of a triple combination of sevoflurane (approximately 0.4 minimum alveolar concentration (MAC), age-corrected), propofol (approximately 2 mg/kg/hour) and remimazolam (approximately 0.3 mg/kg/hour), thereby combining the antiemetic profile of propofol with the depressing (immobilizing) effect on spinal motor neurons of a volatile anesthetic [3] and the hemodynamic stability of remimazolam to create a high-quality general anesthesia by combining the beneficial aspects of the three narcotic agents.

The Institutional Review Board (IRB) (METC Oost-Nederland, file number: 2023-16680) has approved this retrospective case series which only used data already stored in the electronic files of the patients without further interventions or measurements. Only data from patients who gave explicit consent that all their clinical data may anonymously be used for retrospective research were included, as documented in the electronic patient record of each patient. The IRB waived additional patient consent requirements.

## Case presentation

We report five patients who received a combination of sevoflurane, propofol, and remimazolam. The demographic characteristics of these five patients are shown in Table 1.

**Table 1:** Patient characteristics. ETT: endotracheal tube; LMA: laryngeal mask

Characteristics	Patient 1	Patient 2	Patient 3	Patient 4	Patient 5
Age (years)	56	23	23	66	76
Gender (male/female)	Male	Male	Male	Female	female
Height (cm)	161	173	163	172	171
Weight (kg)	67	65	62	90	90
Type of surgery	Vascular	Orthopedic	Orthopedic	Trauma	Trauma
Duration of surgery (minutes)	184	54	53	81	111
Airway	ETT	LMA	LMA	ETT	ETT

Next to the standard non-invasive monitoring (electrocardiogram, oxygen saturation, non-invasive blood pressure), a frontal electroencephalogram (EEG) (bispectral index (BIS)) was measured. At induction, the infusion pumps with propofol (approximately 2 mg/kg/hour) and remimazolam (approximately 0.3 mg/kg/hour) were started and administered via the same intravenous line. A bolus of sufentanil (7.5-10 µg for a laryngeal mask and 20 µg for an endotracheal tube) and a bolus of remimazolam (range: 0.18-0.32 mg/kg) were administered. A single dose of rocuronium was given for placing of the endotracheal tube (not for positioning the laryngeal mask). Sevoflurane was added at approximately end-tidal 0.4 MAC, age-corrected. This age-corrected MAC was directly displayed on the monitor of the GE Datex Ohmeda Advance anesthesia machine. Internally, the age-corrected MAC for sevoflurane was calculated according to the following formula: MAC age = 2.05 × 1.32 × 10^(-0.00303 × patient age in years). After induction, piritramide was given as a long-acting opioid (average 0.1-0.2 mg/kg to reach a respiratory rate of 8-12/minute in the spontaneously breathing patients with a laryngeal mask and based on clinical assessment in the patients with an endotracheal tube) (Table 2). No other opioids were administered after the induction of anesthesia.

**Table 2:** Perioperative medication. The maintenance dose is calculated as the mean dose from the start of surgery to five minutes before the end of surgery. MAC: minimum alveolar concentration

	Medication	Patient 1	Patient 2	Patient 3	Patient 4	Patient 5
For induction	Sufentanil (µg)	20	10	7.5	20	20
	Remimazolam (mg)	13	20	20	16	16
For maintenance	Propofol (mg/kg/hour)	1.95	2.00	1.94	1.67	1.78
	Remimazolam (mg/kg/hour)	0.30	0.31	0.31	0.25	0,27
	Age-corrected MAC sevoflurane	0.44	0.44	0.39	0.41	0.40
Opioids	Piritramide (mg/kg)	0.11	0.08	0.12	0.11	0.17

As is standard in our department, premedication with 1,000 mg paracetamol and 150 mg pregabalin for orthopedic surgery, an intraoperative multimodal concept with non-steroidal anti-inflammatory drugs parecoxib, dexamethasone, and NMDA receptor antagonists (esketamine and magnesium) were used (Table 3).

**Table 3:** Intraoperative multimodal analgesia components. y: given; n: not given; IV: intravenous

Medication	Patient 1	Patient 2	Patient 3	Patient 4	Patient 5
Parecoxib IV (mg)	n	40	40	n	40
Dexamethasone IV (mg)	8	8	8	8	8
Esketamine IV (mg); (mg/hour)	10; 10	15; -	15; -	15; -	5; 5
Magnesium IV (mg); (mg/hour)	n	2,000	2,000	2,000	2,200; 500

All patients remained hemodynamically stable at a sufficient hypnotic depth (BIS 40-60). No patient movement was observed during surgery. No bradycardia, tachycardia, or hypertensive periods occurred. To prevent hypotension, an infusion pump with diluted noradrenaline (10 µg/mL) was started in two patients during the induction of anesthesia (Table 4). No patient received atropine, ephedrine, or phenylephrine.

**Table 4:** Hemodynamic parameters and BIS. BIS, noradrenaline dose, and hemodynamic parameters are calculated as mean ± standard deviation from the start of surgery to five minutes before the end of surgery. Patients 2, 3, and 4 did not receive noradrenaline. BP: blood pressure; HR: heart rate; bpm: beats per minute; BIS: bispectral index score

Hemodynamic parameter	Patient 1	Patient 2	Patient 3	Patient 4	Patient 5
BP (mean) (mmHg)	80.1 ± 5.9	79.5 ± 6.1	66.8 ± 4.7	68.8 ± 4.7	74.1 ± 8.3
BP (systolic) (mmHg)	97.4 ± 6.2	113.1 ± 5.1	117.7 ± 5.6	110.3 ± 6.4	106.8 ± 11.2
BP (diastolic) (mmHg)	73.9 ± 6.3	64.3 ± 8.0	40.9 ± 7.6	48.4 ± 4.1	60.3 ± 6.5
HR (bpm)	64.6 ± 4.9	71.4 ± 3.6	76.9 ± 4.3	55.3 ± 4.1	61.0 ± 5.0
BIS	32.4 ± 5.4	47.8 ± 4.1	33.1 ± 3.4	51.3 ± 6.3	46.6 ± 5.4
Noradrenaline (µg/kg/minute)	0.035	0	0	0	0.017

In the last five minutes before the end of surgery, sevoflurane and the infusions of remimazolam and propofol were gradually reduced until stopped. The documented time between the end of anesthesia and extubation was between one and six minutes.

## Discussion

A combination of sevoflurane, propofol, and remimazolam was used successfully in these five patients. Although combining drugs for hypertension or chemotherapy is quite standard, it is not yet used as standard practice for hypnotic drugs in anesthesia. Wolf et al. compared a combined intravenous-volatile anesthesia (propofol plus volatile anesthetic) to intravenous or volatile anesthesia alone in a systematic review and meta-analysis [2]. They found a significantly reduced risk for PONV for combined anesthesia compared to volatile anesthesia and a significantly reduced movement during surgery for combined compared to total intravenous anesthesia.

Petkus et al. described the combined use of continuous propofol 3 mg/kg/hour and continuous remimazolam 0.3-0.42 mg/kg/hour in a recent case report of a pediatric patient with a suspected family history of malignant hyperthermia [4].

Yang et al. combined inhaled desflurane 0.3 age-adjusted MAC with continuous intravenous infusion of remimazolam (median: 0.36 mg/kg/hour) in 147 patients aged more than 60 years in a study of postoperative delirium in older adult patients undergoing orthopedic surgery [5].

To our knowledge, a triple combination of sevoflurane, propofol, and remimazolam has not been reported yet. While other dosing schemes are possible, our dosing scheme of 0.4 age-corrected MAC sevoflurane plus 2 mg/kg/hour propofol plus 0.3 mg/kg/hour remimazolam may serve as a starting point. We calculated the used dose of propofol and remimazolam based on the actual weight (Table 1). Others might prefer calculating the dose based on the ideal weight. The age-corrected MAC sevoflurane was displayed on the anesthesia monitor during anesthesia. The GE Datex anesthesia machine uses the formula of Eger [6] with an assumed MAC of 2.05 for sevoflurane. Other formulas, e.g., that of Mapleson [7] have alternatively been used. We reported the time from the end of surgery to extubation, but further data about the quality of recovery after anesthesia were incomplete and therefore not reported in this case series, such as data on postoperative pain scores or PONV. Nonetheless, the electronic patient files revealed that none of the patients received antiemetic drugs postoperatively in the recovery room or the ward and all patients recovered uneventfully.

The BIS was recorded but not used for further fine-tuning. As no patient in this case series approached states of too light/superficial anesthesia, EEG monitoring does not seem to be essential for this form of triple anesthesia and could be omitted in future patients. Alternatively, a personalized optimization of the proposed dosing regimen based on the actual BIS value is possible.

Using methadone, due to its slower pharmacokinetic and inherent NMDA receptor antagonism, instead of piritramide might further improve intraoperative hemodynamic stability and postoperative pain scores [8].

All the drugs were given via the same intravenous line. Kondo et al. conducted physical compatibility tests and concluded that all tested drugs (remifentanil, fentanyl, rocuronium, dexmedetomidine, and midazolam) were physically compatible with remimazolam during simulated Y-site administration [9]. Hofmann et al. also tested multiple drugs for possible precipitation with remimazolam in a simulated Y-site administration [10]. They found no precipitation with remimazolam, e.g., esketamine, intravenous magnesium, noradrenaline, ondansetron, piritramide, and rocuronium.

Most impressive in this case series seems the high stability of anesthesia, in terms of hemodynamic stability, only using very low doses of noradrenaline in two patients. In both patients, noradrenaline was already started during anesthesia induction to prevent hypotension. Given the very low dose of noradrenaline intraoperatively (Table 4) and the relative high average mean blood pressure of 80 and 74 mmHg in the two patients who received intraoperative noradrenaline with a lowest intraoperative mean blood pressure of 68 mmHg in patient #1 and 62 mmHg in patient #5, it may be questioned whether intraoperative noradrenaline was needed in these two patients. Nonetheless, the withdrawal of sympathetic tone with the induction of anesthesia and the vasodilating properties of anesthetic drugs commonly results in a certain degree of lowered blood pressure intraoperatively with the insurmountable need for antihypotensive drugs in some patients.

In addition, the sufficient depth of anesthesia, with a uniform dosing regimen for all patients without the need for a single change of infusion speed for the propofol and remimazolam infusion pumps and no intended increase or decrease of the end-tidal sevoflurane concentration and no extra opioid after the administration of the long-acting opioid piritramide post-induction. This may be considered a big step forward toward minimal intervention (autopilot) anesthesia.

## Conclusions

Based on the results of this case series, we can conclude that triple anesthesia with propofol, sevoflurane, and remimazolam provides stable anesthesia with sufficient anesthetic depth, highly hemodynamic stable anesthesia, a uniform dosing regimen for all patients and provides a big step forward toward autopilot anesthesia. However, the concept of triple anesthesia with sevoflurane, propofol, and remimazolam needs further research.
